# Supplementary material for: Gut–Brain Axis: Insights from Hippocampal Neurogenesis and Brain Tumor Development in a Mouse Model of Experimental Colitis Induced by Dextran Sodium Sulfate
Source: Int J Mol Sci. 2022 Sep 29;23(19):11495. doi: 10.3390/ijms231911495 (PMC9569494; doi:10.3390/ijms231911495)
Supplement: Supplementary file 1 [file ijms-23-11495-s001.zip › ijms-1919592-supplementary.pdf]

Supplementary Section S1

Assessment of DSS concentration to induce colitis

A pilot study to determine the appropriate concentration of DSS (molecular mass 36,000-50,000 Da, Cayman Chemical Company, Ann Arbor, USA) to induce colitis was carried out in six experimental mouse groups (n = 5). Each mouse group was treated with a decreasing concentration of DSS (2.5%, 1.5%, 1%, 0.7%, 0.5%) dissolved in autoclaved drinking water, for 7 days and one control group was treated with regular drinking water. Survival and body weight were monitored for 14 days. Results indicated a mortality close to 100% for DSS doses higher that 0.7% while 0.5% determined a 100% survival rate, but only one mouse to showed a mild body weight reduction.

DSS %	Survival %	Death %	Responder %	Mild Responder %	No responder %
2.5%	0%	100% (n=5)	100% (n=5)	0%	0%
1.5%	0%	100% (n=5)	100% (n=5)	0%	0%
1.0%	20% (n=1)	80% (n=4)	100% (n=5)	0%	0%
0.7%	80% (n=4)	20% (n=1)	50% (n=2)	50% (n=2)	0%
0.5%	100% (n=5)	0%	0%	20% (n=1)	80% (n=4)

Weight loss percentage >25% caused mouse death. Mice showing a body weight loss comprises between 25% and 10%, were considered "responder", those showing a weight loss between 10% and 1%, "mild responder" and mice showing any weight loss <0% "no responder".

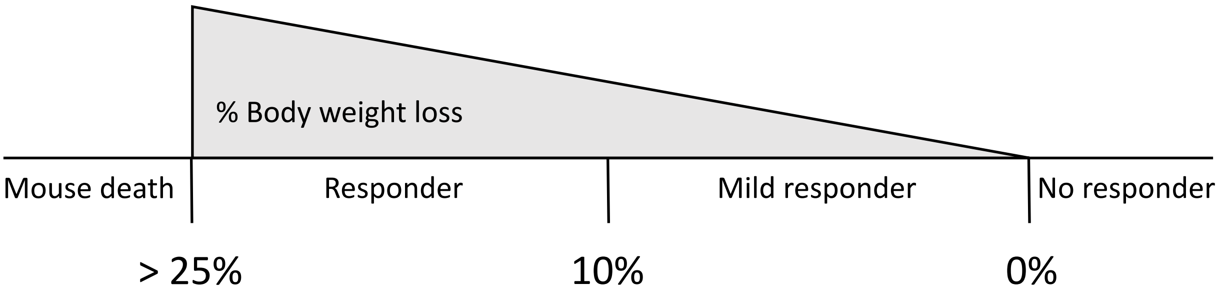

With DSS at 0.7% we had a mouse survival-rate of 80%, although only 50% of the survivors were "responders" and 50% were "mild responders". The histopathological analysis of the "mild responders", however, confirmed the presence of damage in their colon, and we have therefore select 0.7% as the optimal concentration for the induction of acute and chronic colitis.
